# **Cerebral vasculitis caused by *Talaromyces marneffei* and *Aspergillus niger* in a HIV**-**positive patient: a case report and literature review**

**DOI:** 10.1007/s13365-021-01032-5

**Published:** 2022-01-03

**Authors:** Yidong Gao, Man Qu, Chao Song, Lufeng Yin, Min Zhang

**Affiliations:** 1 Department of Encephalopathy, The Third People’s Hospital Affiliated to Fujian University of Traditional Chinese Medicine, Fujian 350122 Fuzhou, China; 2 The State Key Laboratory of Translational Medicine and Innovative Drug Development, Jiangsu Simcere Diagnostics Co. Ltd., Nanjing, 210042 China

**Keywords:** Cerebral vasculitis, *Talaromyces marneffei*, *Aspergillus niger*, Metagenomic next generation sequencing, Human immunodeficiency virus

## Abstract

Cerebral vasculitis is a long-standing but flourishing and fadeless research topic. Infections are a frequent cause of cerebral vasculitis, vital to diagnose due to involvement of specific anti-infection treatments. A 65-year-old man visited the hospital for his neurological symptoms without obvious inducements. After admission, radiological examination and comprehensive conventional microbiological tests (CMTs) revealed suspected intracranial infectious vasculitis. Metagenomic next-generation sequencing (mNGS) and reverse transcription-polymerase chain reaction further confirmed that his cerebral vasculitis was caused by *Talaromyces marneffei* (*T. marneffei*) and *Aspergillus niger* (*A. niger*) co-infection. The patient’s final diagnosis changed from initial herpetic encephalitis, due to the past history of cephalosome and facial herpes and non-significant antiviral therapeutic effects, to fungal cerebral vasculitis. The patient was discharged after use of targeted antifungal therapies on day 18 of his admission, and his associated symptoms disappeared completely at follow-up 3 weeks later. We first illustrated the presence of uncommon cerebral vasculitis caused by *T. marneffei* and *A. niger* in a human immunodeficiency virus-positive patient. In clinically suspected patients with infectious cerebral vasculitis, mNGS should be performed to detect potential pathogens if CMTs may not provide useful pathogenic clues, highlighting the importance of mNGS in the diagnosis and treatment of infectious diseases.

## Introduction

Cerebral vasculitis is defined as inflammation occurring associated with destructive changes, occlusion, and infarction within the wall of intracranial blood vessels(Kraemer and Berlit 2021; Camuset et al. 2012). The prognosis of cerebral vasculitis, including infectious cerebral vasculitis, is closely related to early recognition and diagnosis due to its severity(Blancart et al. 2021; Wu et al. 2021). Thus, the key to understand infectious cerebral vasculitis is to get a good grasp of its underlying pathogens. However, conventional microbiological tests (CMTs) have competitive weaknesses, such as long turnaround time, complex process, low positive rate, and throughput. Metagenomic next-generation sequencing (mNGS), by contrast, directly detects the nucleic acid sequence of microorganisms in clinical specimens, skipping microbial culture (Gu et al. 2019; Alzahrani et al. 2021; Zhang et al. 2020), and covers a variety of pathogenic microorganisms including viruses, bacteria, fungi, and parasites quickly and objectively(Gu et al. 2019; Petersen et al. 2020). Here, we exemplified an old man who had clinically suspected viral encephalitis and was ultimately diagnosed as fungal cerebral vasculitis using mNGS. Additionally, this was the first case to report the presence of cerebral vasculitis caused by *T. marneffei* and *A. niger* in human immunodeficiency virus (HIV)-positive patient.

## Case report

A 65-year-old man without obvious inducements was hospitalized for slow response, decreased speech and dysphagia. Two days ago, he went to the local hospital, and his brain computed tomography (CT) examination indicated cerebral infarction with a small amount of oozing blood. To seek for further diagnosis and treatment, he was transferred to our hospital with suspected herpetic encephalitis or arteriosclerotic cerebral infarction, considering that the patient had a past history of cephalosome, facial herpes, and type-2 diabetes. His blood pressure was 109/65 mmHg, pulse was 103 bpm, respiration was 20 bpm, and temperature was 36.8 °C on admission. Cerebrospinal fluid was obtained by lumbar puncture after admission, and then antiviral therapies (Acyclovir, 500 mg, every 8 h) were given.

Laboratory examination revealed increased neutrophil percentage of 89.5% (normal 51–75), absolute neutrophil cell counts of 7.58 × 10^9^/L (normal 2.0–7.0), C-reactive protein of 81.7 mg/L (normal 0–10), D-Dimer 9.42 μg/mL (normal 0–1), glycosylated hemoglobin of 6.20 mmol/L (normal 4–6), and erythrocyte sedimentation rate 44.0 mm/h (normal 0–15). Meanwhile, a set of autoimmune antibody tests, including anti-U1RNP, anti-SSA, anti-Sm, anti-Slc-70, antinuclear antibody, anti-ribosomal P protein antibody, anti-SSB, and anti-J01, all indicated negative, which excluded the diagnosis of encephalitis caused by autoimmune. Other functional tests were within the normal range, except for HIV antibody positivity.

**Fig. 1:**
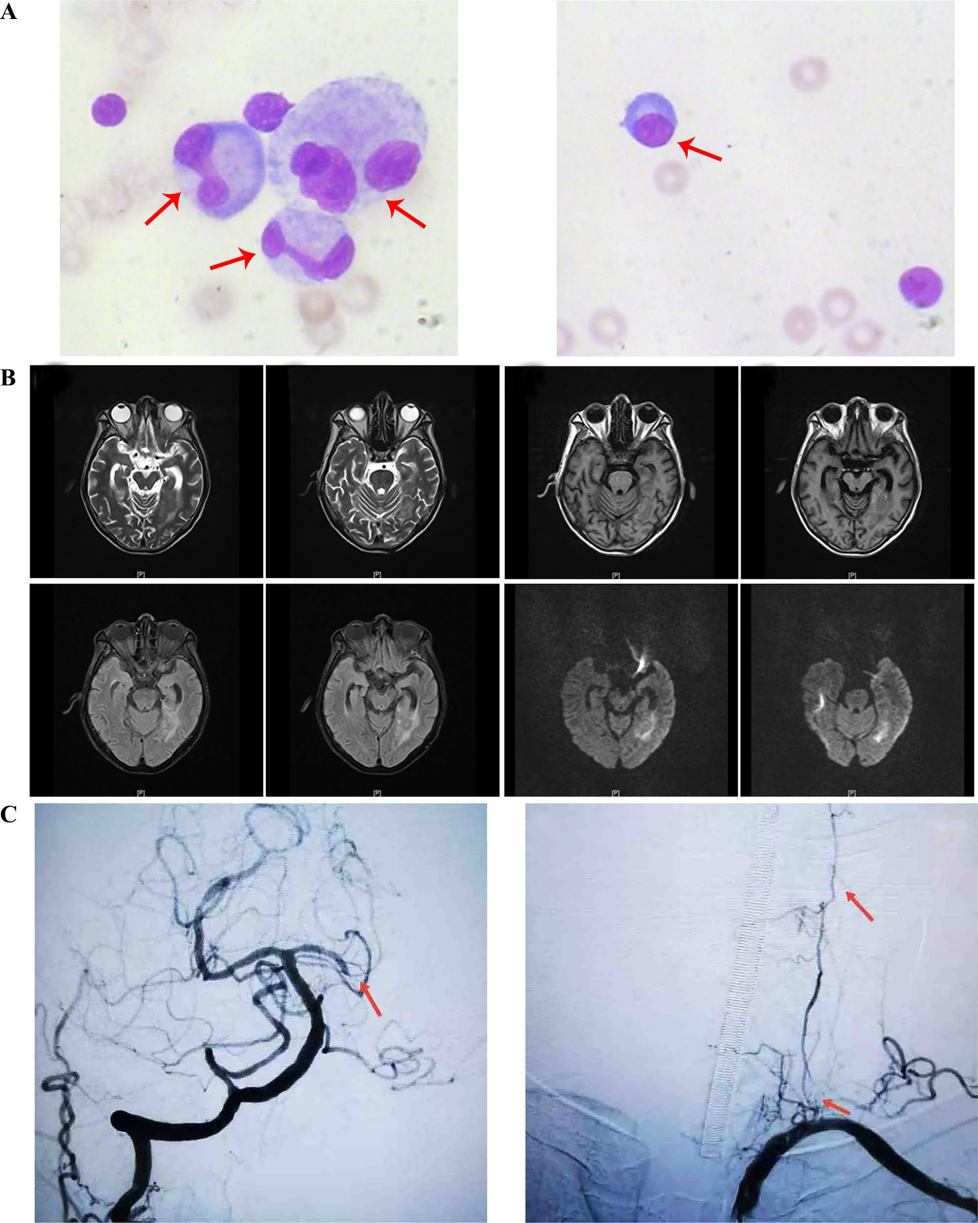
Results of imaging and cerebrospinal fluid cytology examinations. (**a**) Image of cerebrospinal fluid cytology examination. Active monocytes and plasmacytes from left to right were indicated by the red arrow. (**b**) Brain MRI results. These images were in pairs from left to right and from top to bottom, and represented T1, T2, Flaire, and dwi, respectively. (**c**) Cerebral angiography examination. Abnormal signals were indicated by the red arrow

The cerebrospinal fluid cytology examination indicated that the patient’s white blood cell counts (15 × 10^6^/L; normal 0–8), red blood cell counts (480 × 10^6^/L; normal 0), total protein (2474.0 mg/L; normal 200–450), and lactate dehydrogenase (111.10 U/L; normal 3–40) levels significantly raised, while glucose level assay (1.93 mmol/L; normal 2.5–4.5) decreased. Cytological assay of his cerebrospinal fluid exhibited abnormal signs in the percentage of active monocytes (18%) and plasmacytes ( +) (Fig. 1a). Cerebrospinal fluid culture was negative. After admission, the brain magnetic resonance imaging (MRI) showed hemorrhagic infarction in the temporal lobe, which implied intracranial infectious vasculitis may occur (Fig. 1b). In order to confirm the diagnosis, we performed whole brain angiography on the seventh day after admission. It showed that the patient had multiple lesions in the cerebral artery, mainly in the posterior circulation. The initial part of the left vertebral artery was indistinct, the distal vessels were thin, and there was no progression far from V2. The right vertebral body was dominant, and the left posterior cerebral artery was occluded far away from P1 segment. The diagnosis of cerebral vascular inflammatory lesions was confirmed (Fig. 1c).

**Fig. 2:**
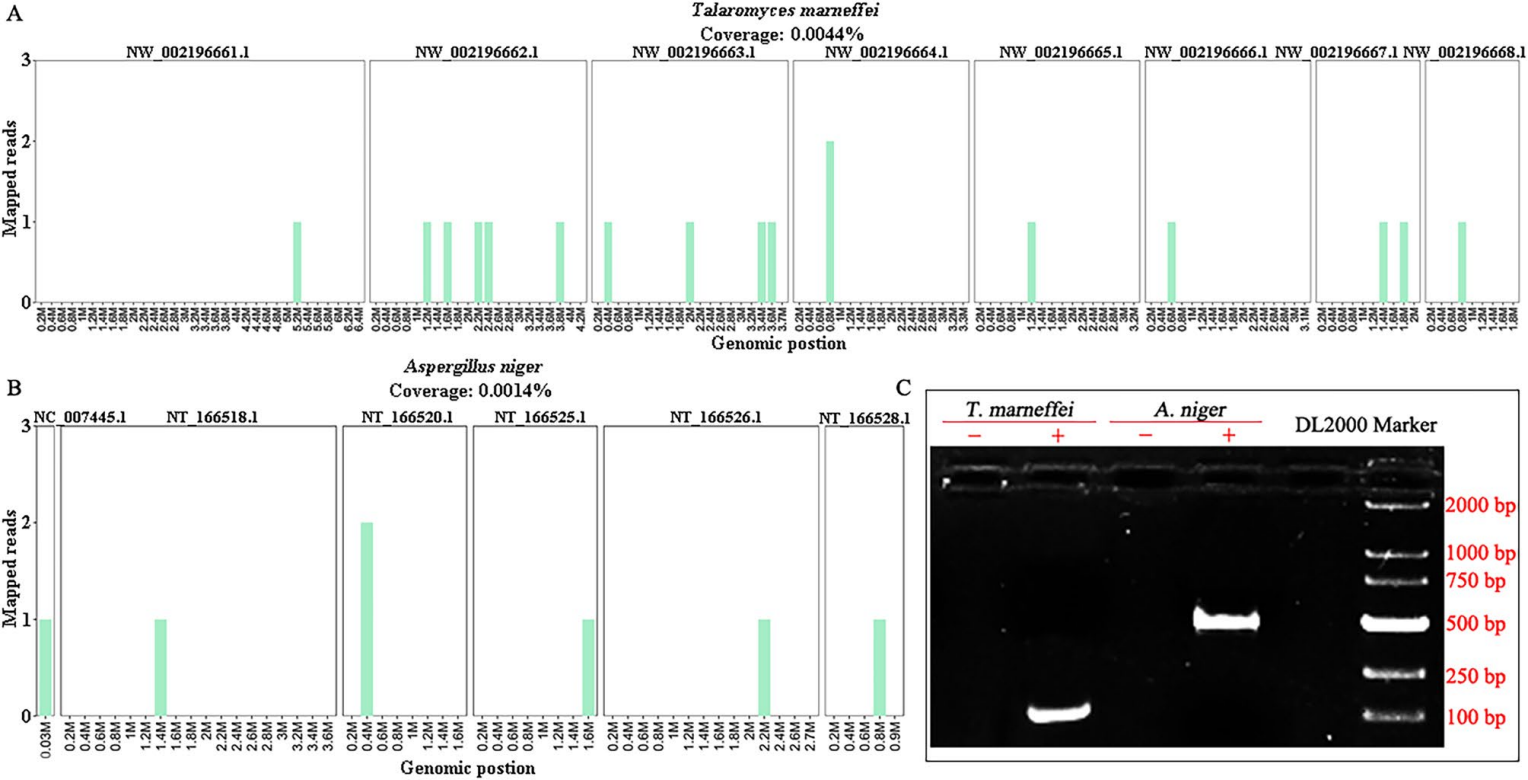
Molecular diagnosis of *T. marneffei* and *A. niger*. (**a**) Mapped reads of *T. marneffei* by the means of mNGS. (**b**) Mapped reads of *A. niger* by the means of mNGS. (**c**) RT-PCR confirmation test

On day 3 of admission, the patient had thick sputum elimination with high fever (38.7℃). Lung CT examination showed that his physical condition worsened with pulmonary infection. And the patient’s sputum culture was examined, but no significantly positive pathogen culture was identified. Intravenous injection of cefotaxime sodium at a dose of 2 g per 12 h was implemented to deaden his pulmonary infections empirically. Concurrently, mNGS was performed in SimcereDx Lab (Nanjing, China) to identify potential pathogens in cerebrospinal fluid due to disease progression. mNGS revealed 13 T*. marneffei* and 9 *Aspergillus niger* specific sequences with reads coverage of 0.0044% and 0.0014%, respectively (Fig. 2a, b). The following reverse transcription-polymerase chain reaction (PCR) test confirmed the pathogenic results of mNGS (Fig. 2c). Primer information of *T. marneffei* (101 bp): forward primer: 5-TTCCCGAGCGAGTGACAGA − 3 and reverse primer 5-GCTTGTGTGTTGGGTGTGGT −3 and *A. niger* (500 bp): forward primer: 5-GGG CAA AGGGTTGGGTCTTC −3 and reverse primer 5′ GACGAGGACGGCACGAGGA −3. Thus, his final diagnosis was adjusted to fungal intracranial vasculitis caused by *T. marneffei* and *A. niger*. And at the same time, his treatment options were changed into intravenous injection of amphotericin B from 3 mg on day 1, 5 mg on day 2, until 40 mg on day 10. In order to prevent the side effects, a small dose of dexamethasone 5 mg was given before use.

On day 10 of admission, high fever disappeared. On day 13, the lung CT examination showed significant remission of the local focus caused by pulmonary infection. The patient’s symptoms improved and discharged on day 18 of admission after a long-term anti-infection. After leaving hospital, he was further treated with itraconazole 200 mg per 12 h. At follow-up 3 weeks later, the fever and neuronal disorders vanished completely.

## Discussion

In this case, considering that the patient had a history of facial herpes and was not cured on his hospitalization, and the brain CT showed the temporal lobe lesion with hemorrhage, herpetic encephalitis was considered for pre-diagnosis; hence antiviral treatment was given. CMT results were negative for autoimmune encephalitis caused by HIV positivity. Genetically pathogenic analysis of *T. marneffei* and *A. niger* positive in cerebrospinal fluid excluded the possibility of herpetic infection-derived cerebral vasculitis. Meanwhile, cerebral angiography showed occlusion in the left posterior cerebral artery and hemorrhagic infarction lesions in the bilateral medial temporal lobe. Collectively, the diagnosis was finally adjusted to fungal cerebral vasculitis. Apart from genetic evidence, the curative effect of targeted antifungal therapies and 3-week follow-up reaffirmed the diagnosis of fungal cerebral vasculitis.

Fungal infections are one of the major causes of adverse clinical outcomes in cerebral vasculitis worldwide (Armstrong-James et al. 2014; Gullo 2009). Since the first case of fungal cerebral vasculitis described by Rao, V.R.K. et al. a series of cases have been reported (Rao et al. 1978a). A systematic review of the fungal cerebral vasculitis was retrieved from Web of Science Core Collection, and up to May 28th, 2021, 20 papers had been published in total (Table 1). Fungal cerebral vasculitis frequently occurs in patients with severe cellular immunosuppression (hematopathy, transplantation, HIV infection, and immunosuppressive therapy), with a dismal prognosis (Jung et al. 2018; Leroy et al. 2020; Ermak et al. 2014; Eucker et al. 2000; Van Rooij et al. 2018). Infection-derived vasculitis is caused by angiogenic pathogens, including *Aspergillus sp.*, *Candida albicans*, *zygomycotina sp.*, *Cryptococcus neoformans,* and so on, and their etiopathogenesis can be summarized as follows: subarachnoidal meningitis, sinuses or orbital infection, and immune mechanism in the context of chronic infections (Lampros et al. 2021; Herlin et al. 2015; Thirunavukkarasu et al. 2021). Early recognition and diagnosis of cerebral vasculitis can, to a great extent, alleviate the risk of its severity and improve prognosis (Rao et al. 1978a; Jung et al. 2018; Salvarani et al. 2007; Sasaki et al. 2010; Ellis et al. 2018).

**Table 1:** A systematic review of the literature on fungal cerebral vasculitis

References	Patient’s information		Detection methods	Etiopathogenesis	Length of stay	Clinical outcomes
	Age (year)	Sex			(days)	
(Rao et al. 1978b)	20	-	Histological examination	*Mucor sp.*	-	Recovery
(Ho and Allevato 1986)	41	Female	Microscopic examination	*Paecilomyces javanicus*	15	Died
(Lee et al. 1996)	47	Female	Histological examination	*Mucor sp.*	6	Died
(Grimes et al. 1998)	37	Female	Bacterial culture	*Candida albicanss*	69	Died
(Erly et al. 1999)	33	Male	Histological examination	*Coccidioides immitis*	9	Died
	74	Male	Bacterial culture		14	Died
(Eucker et al. 2000)	18	Female	Bacterial culture	*Absidia corymbifera*	6	Died
(Rickert et al. 2002)	10	Female	Histological examination	*Mucor sp.*	21	Died
(Roberts et al. 2004)	71	Female	Histological examination	*Aspergillus sp.*		Died
(Laurencikas et al. 2006)	12	Male	Aspergillum antigen test	*Aspergillus sp.*	31	Died
(Marazzi et al. 2008)	5	Male	Histological examination	*Candida albicans*	8	Died
(Sasaki et al. 2010)	35	Female	Histological examination	*zygomycotina sp.*	115	Died
		Female	Tissue pathology examination		-	Recovery
	68	Female	Bacterial culture		28	Died
(Martins et al. 2010)	56	Female	Bacterial culture	*Aspergillus sp.*	-	Died
(Ermak et al. 2014)	57	Female	Tissue pathology examination	*Mucor sp.*	2	-
(Moore et al. 2016)	76	Male	Gomori methenamine silver stain	*Aspergillus sp.*	1	Died
(Ellis et al. 2018)	38	Male	Bacterial culture	*Cryptococcus neoformans*	19	Recovery
(Jung et al. 2018)	29	Female	Bacterial culture and serologic testing	*Aspergillus fumigatus*	31	Recovery
(Buchanan et al. 2019)	26	Male	Serologic testing	*Coccidioides immitis*	31	Partial recovery
(Ueno et al. 2019)	60	Female	Histological examination	*Aspergillus sp.*	48	Died
(Leroy et al. 2020)	61	-	bacterial culture and qPCR	*Aspergillus* *fumigatus*	-	Recovery
(Polk et al. 2020)	26	Male	CSF cryptococcal Ag test and bacterial culture	*Cryptococcus neoformans*	36	Partial recovery
Our study	65	Male	mNGS and RT-PCR	*Talaromyces marneffei* and *Aspergillus niger*	18	Recovery

In this case, *T. marneffei* and *A. niger* were genetically verified as infectious causes of vasculitis using mNGS and RT-PRC. *T. marneffei* usually causes invasive fungal infection, characterized by lack of specificity in clinical presentation and difficulties in diagnosis (Chan et al. 2016). *A. niger*-derived invasive pulmonary aspergillosis is one of the most common mould infections in inpatients (Schmiedel and Zimmerli 2016). Both two strains primarily result in respiratory and bloodstream infections, and few cases have been described in cerebral infection, not to mention the co-infection with *T. marneffei* (Chan et al. 2016; Schmiedel and Zimmerli 2016).

## Conclusion

We first reported the presence of cerebral vasculitis caused by *T. marneffei* and *A. niger* in a HIV-positive patient. By summarizing the literature on fungal cerebral vasculitis, the importance of pathogenic identification was further emphasized in the early stage. Briefly, our case highlights the crucial role of mNGS in identification of specific pathogens and provides a new insight into the diagnosis of infectious cerebral vasculitis.

## Data Availability

Data supporting the research results of this study can be obtained on request from the corresponding authors Dr. Zhang.
